# Changes in the deep subsurface microbial biosphere resulting from a field-scale CO_2_ geosequestration experiment

**DOI:** 10.3389/fmicb.2014.00209

**Published:** 2014-05-14

**Authors:** Andre Mu, Chris Boreham, Henrietta X. Leong, Ralf R. Haese, John W. Moreau

**Affiliations:** ^1^School of Earth Sciences, Faculty of Science, University of MelbourneMelbourne, VIC, Australia; ^2^Department of Microbiology and Immunology, University of Melbourne, Peter Doherty Institute for Infection and ImmunityMelbourne, VIC, Australia; ^3^Cooperative Research Centre for Greenhouse Gas TechnologiesCanberra, NSW, Australia; ^4^Geoscience AustraliaCanberra, NSW, Australia

**Keywords:** CO_2_ sequestration, deep subsurface, supercritical CO_2_, microbial response, CODH, biofilms

## Abstract

Subsurface microorganisms may respond to increased CO_2_ levels in ways that significantly affect pore fluid chemistry. Changes in CO_2_ concentration or speciation may result from the injection of supercritical CO_2_ (scCO_2_) into deep aquifers. Therefore, understanding subsurface microbial responses to scCO_2_, or unnaturally high levels of dissolved CO_2_, will help to evaluate the use of geosequestration to reduce atmospheric CO_2_ emissions. This study characterized microbial community changes at the 16S rRNA gene level during a scCO_2_ geosequestration experiment in the 1.4 km-deep Paaratte Formation of the Otway Basin, Australia. One hundred and fifty tons of mixed scCO_2_ and groundwater was pumped into the sandstone Paaratte aquifer over 4 days. A novel U-tube sampling system was used to obtain groundwater samples under *in situ* pressure conditions for geochemical analyses and DNA extraction. Decreases in pH and temperature of 2.6 log units and 5.8°C, respectively, were observed. Polyethylene glycols (PEGs) were detected in the groundwater prior to scCO_2_ injection and were interpreted as residual from drilling fluid used during the emplacement of the CO_2_ injection well. Changes in microbial community structure prior to scCO_2_ injection revealed a general shift from *Firmicutes* to *Proteobacteria* concurrent with the disappearance of PEGs. However, the scCO_2_ injection event, including changes in response to the associated variables (e.g., pH, temperature and salinity), resulted in increases in the relative abundances of *Comamonadaceae* and *Sphingomonadaceae* suggesting the potential for enhanced scCO_2_ tolerance of these groups. This study demonstrates a successful new *in situ* sampling approach for detecting microbial community changes associated with an scCO_2_ geosequestration event.

## Introduction

The injection of supercritical CO_2_ (scCO_2_) into deep aquifers for long-term storage (geosequestration) is currently being evaluated as a strategy for reducing global atmospheric CO_2_ levels or mitigating industrial CO_2_ emissions (http://www.globalccsinstitute.com/projects/browse). One of the questions that needs to be addressed is the potential response of the subsurface microbial biosphere to increased CO_2_ levels, as microbes are known to inhabit the Earth's crust to depths of approximately three kilometers (Stetter et al., 1993; Chivian et al., 2008) and play a major role in subsurface carbon cycling (Chapelle et al., 2002; Hubert et al., 2011; Bordenave et al., 2012). Geochemical models of the injection of large volumes of scCO_2_ predict significant decreases in pH through the increased formation of carbonic acid that should significantly affect microbial diversity in ways of selecting for growth, or allowing only the survival, of acid tolerant species (Fierer and Jackson, 2006). Similarly, the reaction of scCO_2_ or carbonic acid with aquifer minerals or dissolved ions could result in mineral dissolution or precipitation, leading to changes in ionic strength or the distribution of bioavailable terminal electron acceptors (e.g., ferric iron), that might subsequently affect microbial metabolism (McMahon and Chapelle, 1991; Cozzarelli et al., 1994; Banfield et al., 1999; Rogers and Bennett, 2004). Changes in subsurface microbial community structure or activity could alter terminal electron accepting processes (TEAPs) to impact the geochemistry and mineralogy (Gadd, 2010) of the CO_2_ storage aquifer, potentially resulting in changes to porosity that could impact the distribution of injected scCO_2_. Such changes could also include increased methanogenesis from dissolved CO_2_ (i.e., HCO^−^_3_) under circumneutral pH conditions (Sato et al., 2013), or the CO_2_-driven inhibition of carbon monoxide (CO) oxidation with deleterious impacts to microbial autotrophy and acetogenesis (Ragsdale, 2004).

Previous studies demonstrate that microbes can grow under environmental conditions representative of scCO_2_ storage aquifers, and that certain microorganisms can tolerate short periods of scCO_2_ stress if growing within a biofilm (Ross and Bickerton, 2002; Cunningham et al., 2003, 2009; Mitchell et al., 2009). However, current knowledge of how microbial communities respond to scCO_2_ and related geochemical changes is restricted to *in vitro* experimental studies of representative geological materials, where observations are limited to laboratory-scale effects (Mitchell et al., 2008, 2009; Kirk et al., 2013). In these experiments, however, sustained biological activity after scCO_2_ stress and related changes to the experimental milieu allude to the potential for microbially-driven impacts on CO_2_ storage aquifers that may affect the potential for scCO_2_ geosequestration.

In this study, we characterized the *in situ* microbial community structure and diversity at the 16S rRNA gene level with 454 pyrosequencing (Ronaghi et al., 1996, 1998; Parameswaran et al., 2007) during a scCO_2_ injection experiment at the Otway Basin CO2CRC site (Paterson et al., 2013; www.co2crc.com.au). The aim of this study was to determine the effects of scCO_2_ injection, and its associated derivatives, on the structure of the microbial community. In the conditions of the Paaratte Formation aquifer, the microbial community was hypothesized to be of lower diversity and dominated by thermophilic anaerobes. We also hypothesize that a decrease in biodiversity would occur as a result of the injection of scCO_2_ (admixed with groundwater). Results showed a first order increase in the relative abundance of certain species post-scCO_2_ injection, and a second order decrease in obligately or facultatively fermentative species associated with a disappearance of residual organic compounds from drilling fluid. These results provide new insights into the structure and activity of the subsurface microbial biosphere under exposure to scCO_2_, and in the context of the engineering required for a geosequestration experiment, that will inform groundwater monitoring, geochemical modeling strategies and *in vitro* bioreactor studies in future scCO_2_ storage experiments.

## Materials and methods

### Groundwater sampling

**Figure 1:**
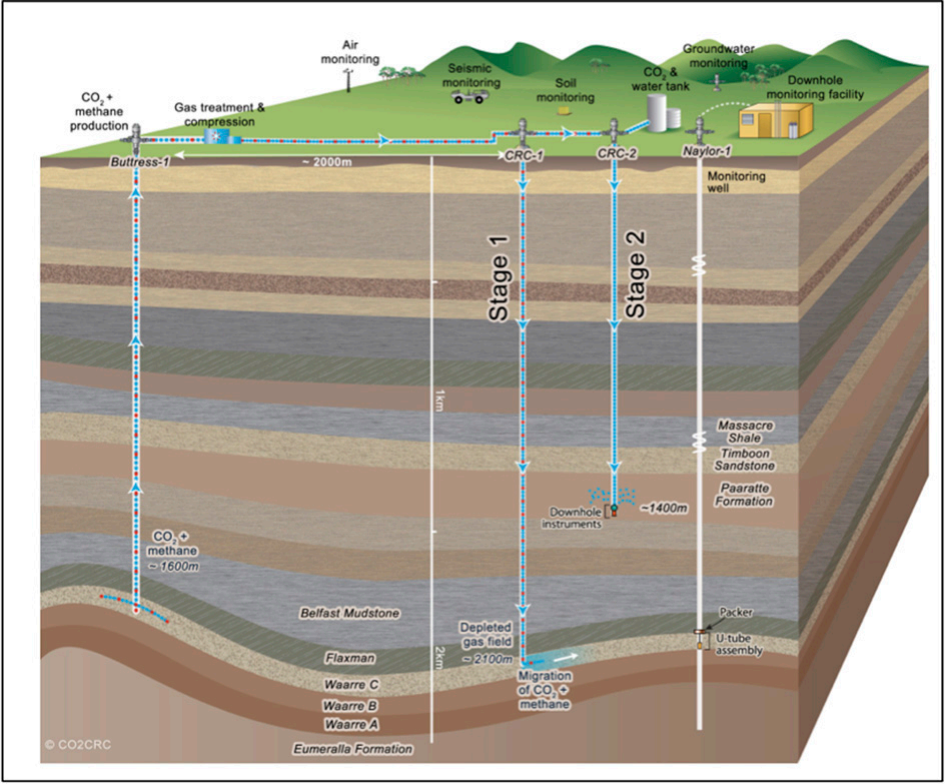
**Graphical representation of the CO2CRC Otway project site**. The sandstone CO_2_ storage aquifer is found within the Paaratte Formation, which is located approximately 1400 m TVDSS at latitude: 38° 31′ 44″ and longitude 143° 48′ 43″. Image source: www.co2crc.com.au.

As part of the Otway Phase 2B field experiment (Paterson et al., 2013), over five hundred tons of ground water were produced during the pre-scCO_2_ injection phase. The respective well was screened at approximately 1400 meters true vertical depth subsea (TVDSS) in a sandstone unit of the Paaratte Formation of the Otway Basin at latitude: 38° 31′ 44″ and longitude: 142° 48′ 43″ (Figure 1). Figure S2B shows the rates of injection and production of formation water and CO_2_ over the experiment, with positive rates corresponding to injection and negative rates to production. To obtain pristine water samples held under *in situ* conditions while not compromising the stability of injected scCO_2_, we utilized a novel, hydraulically sealed “U-tube” sampling system (Freifeld, 2005, 2009) which produced formation water to the surface using a directive flow of high pressure nitrogen gas, under *in situ* pressures of 2010 psi (136.8 atm), in to specialized pressure cylinders. Furthermore, the engineering of the U-tube system allowed for the isolation and storage of 150 ml aliquot of formation water under *in situ* conditions. U-tube water samples were collected for analysis when drilling fluid-derived fluorescein levels declined to 2 × 10^−2^ ppm on the 17th of June 2011.

As shown in Figure S2B, there was the production of 182 tons of formation water (57.7–61.6 days after time origin of the test) subsequent to scCO_2_ injection. Again, a suite of *in situ* water samples was isolated and collected using the U-tube system during this post-scCO_2_ injection period. The frequency of sampling “timepoints” differed between pre- and post-scCO_2_ injection phases due to logistical constraints of different co-occurring experiments involving other research groups (T. LaForce, J. Ennis-King, C. Boreham and L. Paterson, submitted for publication). Samples were recovered once each in the morning and evening during the pre-scCO_2_ injection phase, and at ninety-minute intervals over four consecutive days during the post-scCO_2_ injection phase. Seventy-nine U-tube water samples were collected over the course of the scCO_2_ injection event for baseline geochemical analyses (Figure S3), whilst a selective subset of these U-tube water samples were allowed for further analyses due to the limited sampling efforts (Table 1). Each of the samples collected into the pressure cylinders were designated the nomenclature “PF—”; where “PF” represents *Paaratte Formation*, and “—” represents the number of U-tube samples since time origin.

**Table 1:**
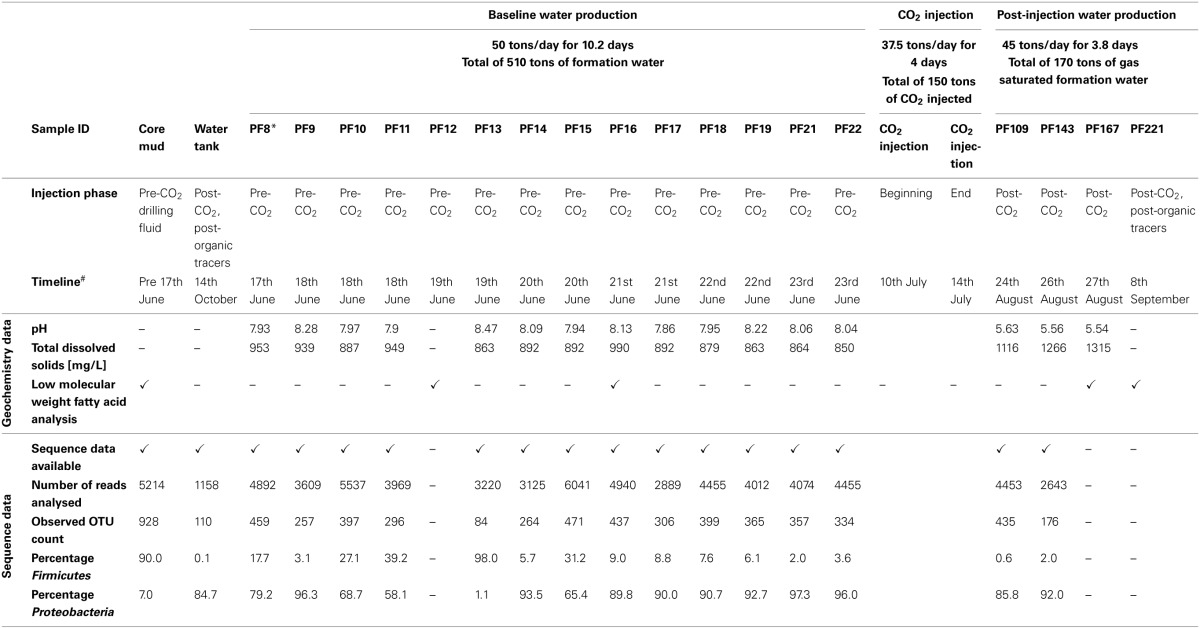
**Summary of the twenty samples analyzed in this study**.

^#^The temporal relationship, as indicated by the day and month, of each sample occurs over the year 2011.

- Data are unavailable due to dedicated sampling time points required by multi-disciplinary collaborative experiments.

^*^PF—; where PF represents “Paaratte Formation,” and—represents the number of U-tube samples since time origin.

### Geochemical analyses

Paaratte Formation water samples were analyzed on site for drilling mud-derived fluorescein concentrations as an indicator for drilling mud contamination using a spectrophotometer (Hach DR/2010) set to a wavelength of 490 nm. When fluorescein levels declined to 2 × 10^−2^ ppm, *in situ* groundwater samples were analyzed on site immediately after collection for pH, temperature, and total dissolved solids (TDS). Thirty-milliliter aliquots were filtered through 0.22 μm filter membranes, and field parameters were measured using a combined Jenway 3540 pH and conductivity meter.

### Low molecular weight fatty acid analysis

Low molecular weight fatty acid extracts were obtained from 100 ml of samples PF12, PF16, PF167, and PF221, and 99 ml of drilling mud fluid using a dichloromethane solvent protocol. Briefly, PF12, PF16, PF167, PF221, and drilling mud fluids were acidified by drop-wise addition of concentrated HCl to an approximate pH of 2. The acidified fluids were transferred to separation funnels where 25 ml of dichloromethane was added. The funnels were held static and unstoppered to allow settling of the mixture after a period of agitation. The denser dichloromethane solutions were then extracted and transferred to 100 ml conical flasks where the process was repeated with another 25 ml of dichloromethane. Approximately 5 grams of sodium sulphate was added to each sample before the flasks were stoppered and held static for 1–2 h.

**Figure 2:**
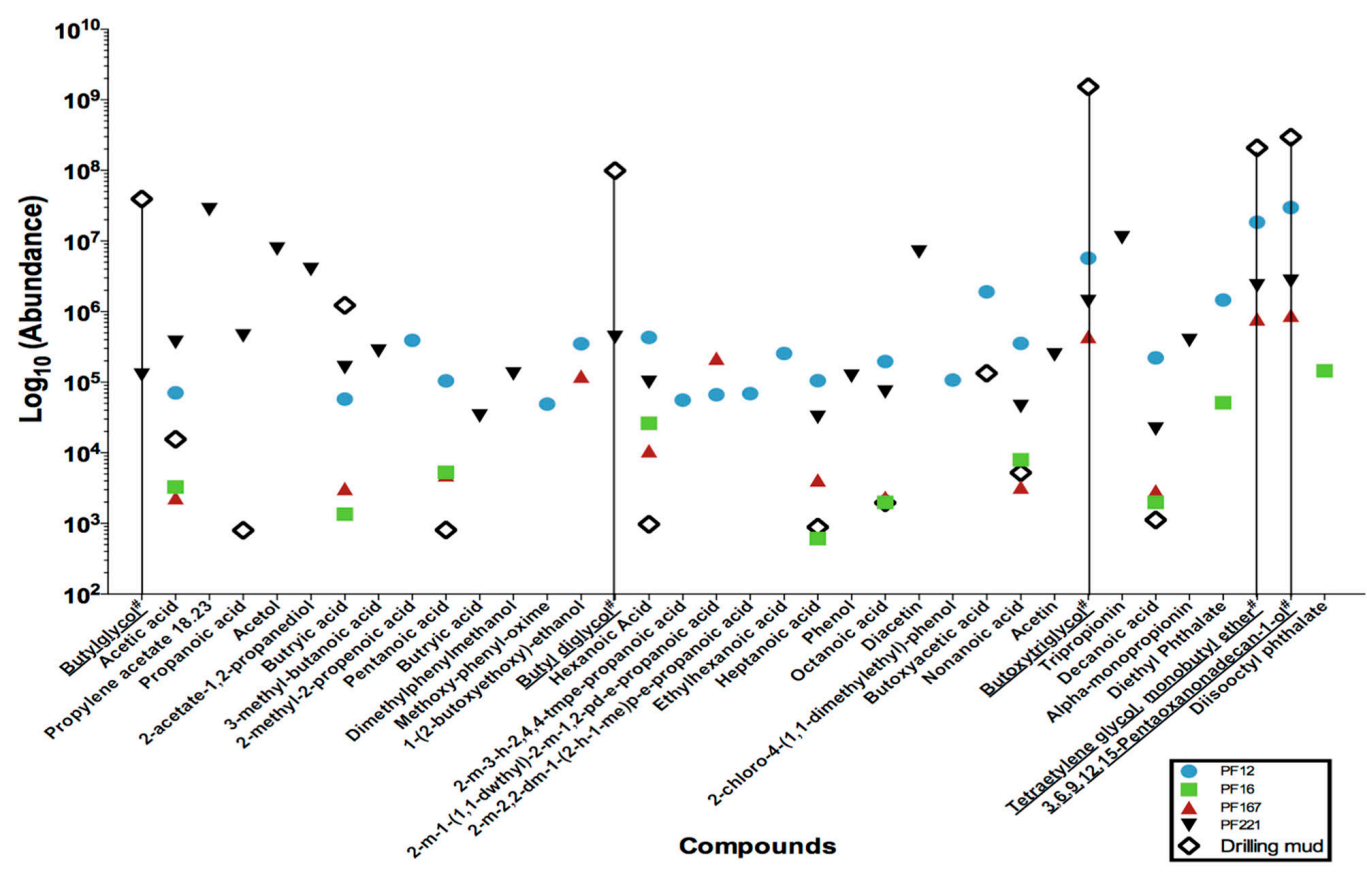
**Low molecular weight fatty acid analyses of a subset of pre-scSCO_2_, post-scCO_2_ injection water samples and drilling mud samples**. Subsets of groundwater samples were assessed for low molecular weight fatty acids by gas chromatography mass spectrometry. (#) Chemical names with an underline represent compounds belonging to the polyethylene glycol group, which is interpreted as residual organic compounds associated with the drilling fluid. A data point on the graph illustrates the presence of that particular compound in the indicated sample. For example, butylglycol was detected in PF221, and drilling mud, but was below the levels of detection in PF12, PF16, and PF167.

The extracts were concentrated using nitrogen gas in a TurboVap LV for an initial period of 15 min at 5 psi and 40°C. Samples were concentrated for 35 to 63 min at approximately 5–7 psi. Gas chromatography mass spectrometry analysis was conducted on an Agilent 5893 Gas Chromatograph equipped with a BP21 nitroterephthalic acid modified polyethylene glycol column of diameter 0.25 mm, length 30 m and film thickness of 0.25 μm. The column was coupled with a HP5973 mass spectrophotometer running at 70 electron volts (eV) in the full scan mode with data acquisition from 10 to 500 atomic mass units. On-column injection of 0.5 μ l of solution to the oven was held isothermally at 40°C for 1 min, and then temperature programmed to increase to 230°C at 4°C min^−1^ increments. The maximum programmed temperature was maintained for 15 min. The helium carrier gas flow was held constant at 1.1 ml min^−1^ throughout the analysis. Absolute concentrations were not determined, as the organic compound composition was not known *a priori* for standard calculations. Instead, the resultant mass spectra were queried against the NIST05 mass spectra library to identify organic compounds and their peaks taken as relative abundances (Figure 2).

### Extraction of whole community genomic DNA

Whole community genomic DNA was extracted onsite within 12 h of sampling. Concentration of biomass by centrifugation was unsuccessful, therefore, pre-scCO_2_ injection water samples were concentrated, as 50 ml aliquots, on to 0.22 μm nylon net filter membranes (Merk Millipore) using vacuum filtration, and processed for genomic DNA extraction using the MoBio Powersoil DNA extraction kit and a modified manufacturer's protocol. The following variations were introduced to the protocol: filter membranes were transferred into MoBio PowerBead tubes, which were secured on to a MP Fastprep-24 beadbeater and processed at 5 Mach per second (M/s) for 45 s. The tubes were pulsed until the filter membranes were completely disrupted. Genomic DNA samples were stored on site at −20°C until further analysis.

Post-scCO_2_ injection water samples were treated in a similar manner. However, given the logistics of the sampling schedule, filter membranes with concentrated biomass for nucleic acid extraction were stored on site in RNAprotect Bacteria Reagent (QIAGEN) at −20°C until processing could be performed in the laboratory. Genomic DNA was also extracted from drilling fluid and water samples obtained from surface water storage tanks (used to hold pre-collected groundwater that was co-injected with scCO_2_). Nucleic acids from these samples are considered controls and represent the exogenous microbial community of peripheral scCO_2_ geosequestration activities (e.g., the use of drilling fluid during the emplacement of injection wells). The control samples thus act as an indicator of the degree of aquifer contamination during our experiment.

### High-throughput barcoded 454 pyrosequencing

Whole community genomic DNA from drilling mud sample 14, surface storage tank 1 water, and U-tube water samples PF8 to PF11, PF13 to PF19, PF21 to PF22, PF109, and PF143, were amplified with native universal small subunit 803 forward and universal SSU1392w reverse (5′-ACG GGC GGT GWG TRC-3′) primers using High-fidelity OneTaq DNA polymerase mastermix (New England Biolabs). SSU803F primer is a combination of 803Fa 5′-TTAGATACCCTGGTAGTC-3′; 803Fb 5′-TTAGATACCCSGGTAGTC-3′; 803Fc 5′-TTAGATACCCYHGTAGTC-3′; 803Fd 5′-TTAGAGACCCYGGTAGTC-3′; in a ratio of 2:1:1:1 for 803Fa:b:c:d. The primer combination of SSU803F and SSU1392wR preferentially amplifies the prokaryotic 16S rRNA gene whilst avoiding the eukaryotic equivalent (Carvalhais et al., 2013). Primers were used at a final concentration of 0.2 μ M under the following thermal cycler conditions: initial denaturation at 94°C for 30 s, thirty cycles of denaturing at 94°C for 30 s, annealing at 55°C for 60 s and extension at 65°C for 36 s, before a final extension at 65°C for 5 min. Amplification was performed using an MJ Research PTC-200 Peltier thermal cycler. Amplicons were sent to the Australian Centre for Ecogenomics (ACE; University of Queensland, Australia) for a secondary 10-cycle amplification using the same primer combination, SSU803F and SSU1392wR, but modified to suit the pyrosequencing chemistry. Furthermore, emulsion PCR and barcoded 454 pyrosequencing (Roche) was performed by ACE following the manufacturer's protocol specific for the GS FLX. The initial amplification was performed to overcome the inefficiencies related to using pyrosequencing-specific primers to amplify low concentration gDNA. The Australian Centre for Ecogenomics has extensively tested the two-step protocol without any unexpected biases. Raw sequence data associated with this study is available from the Sequence Read Archive (SRA) at NCBI under accession number: SRP040950.

### Bioinformatic analyses of 16S rRNA gene sequence data

The QIIME bioinformatics pipeline was employed to analyse sequence data, assign taxonomy, and to determine phylogenetic distributions of each microbial community (Caporaso, 2010). Sequences were quality filtered and demultiplexed using the QIIME module *split_libraries.py*. The quality filtered data were subjected to the QIIME workflow with the default settings (Carvalhais et al., 2013; Dennis et al., 2013). Sequence data were clustered into OTUs at 97% pairwise identity using the UCLUST (Edgar, 2010) seed-based algorithm. A representative sequence from each OTU was aligned using the PyNAST tool (Caporaso et al., 2010) and queried against the Ribosomal Database Project (Wang et al., 2007) for taxonomy assignment. The number of sequences per sample was normalized to a default 75% of the sample with the lowest amount of reads (1158 reads per sample) to address biases from unequal sampling efforts prior to beta-diversity analyses. Beta-diversity analysis was performed to elucidate the similarities between communities using the jackknifed-supported (Quenouille, 1956) confidence values for each microbial community to generate the principal coordinate analysis plot (Figure 5A). A bootstrapped hierarchical clustering Newick formatted tree (Figure 5B) was also generated using the unweighted pair group method with arithmetic mean (UPGMA) algorithm (Michener and Sokal, 1957).

The Maximum likelihood tree was assembled using the multiple sequence alignment file generated by the QIIME workflow running on the Molecular Evolutionary Genetics Analysis 5 (MEGA5) tool (Tamura et al., 2011). Metadata associated with the ML phylogenetic tree was displayed together in the form of a heat map (Figure 6) using the script, *plotTreeData*, which was written for the R platform. The script is publically available from: sourceforge.net/projects/srst/files/otherscripts/plotTreeData.R/download.

## Results

### Fluorescein concentrations

Fluorescein concentrations in groundwater samples illustrated a general decreasing trend as formation water was pumped to the surface (Figure S1A). Fluorescein levels declined to 2 × 10^−2^ ppm at timepoint PF8 and formation water was hence considered geochemically pristine. Throughout the pre-injection phase (Figure S1B) drilling mud fluid-derived fluorescein concentrations remained equal to or less than 2 × 10^−2^ ppm. However, spikes in concentration were observed for timepoints PF14, PF15, and PF16.

### Geochemistry data

An overall increase in TDS was observed from pre- to post-scCO_2_ injection samples. Variations in TDS levels were in the range of 140 mg L^−1^ prior to scCO_2_ injection and as high as 392 mg L^−1^ post-scCO_2_ exposure (Figure S3). Formation pressure increased to a stable 2017 psi (137.25 atm) from baseline readings of 2000 psi (136.09 atm), and *in situ* temperature (Figure S2A) and pH dropped to ca. 54.2°C and 5.6 from 60°C and 8.2, respectively, following injection of scCO_2_. The observed changes in pH were primarily due to the formation of carbonic acid as a consequence of partial scCO_2_ dissolution in to groundwater, and the increase in TDS was most likely related to acid-induced mineral dissolution (Kharaka et al., 2006).

### Low molecular weight fatty acid analysis

Gas chromatography mass spectrometry analysis of low molecular weight fatty acids (Figure 2) revealed that samples PF12, PF167, and PF221 contained polyethylene glycols (PEGs) at similar relative concentrations, which were used in the synthesis of drilling fluid for the emplacement of injection and sampling wells. The C_2_, C_4_–C_10_ *n*-alkyl fatty acids were present in both pre-scCO_2_ samples, PF12 and PF16. However, their relative peak concentrations were between one to two orders of magnitude greater in PF12.

### High-throughput barcoded 454 pyrosequencing data

To understand the organic geochemical data in the context of potential microbial impacts, whole community 16S rRNA gene profiling of the groundwater microbial community in the Paaratte Formation was conducted; allowing for the understanding of the potential geochemical impacts, at the phylogenetic level, of scCO_2_ injection on microbial diversity and *in situ* capacity for biogeochemical cycling.

#### Rarefaction curves

Rarefaction analysis of sequence data (Figure S4) illustrated an increasingly asymptotic trend in the number of observed species, as a function of sequences per sample for all microbial communities analyzed. The water tank community was the most diverse with 637 different operational taxonomic units (OTUs) compared to only 78 different OTUs for PF13 at 2852 sequences per sample.

#### Phyla summary

**Figure 3:**
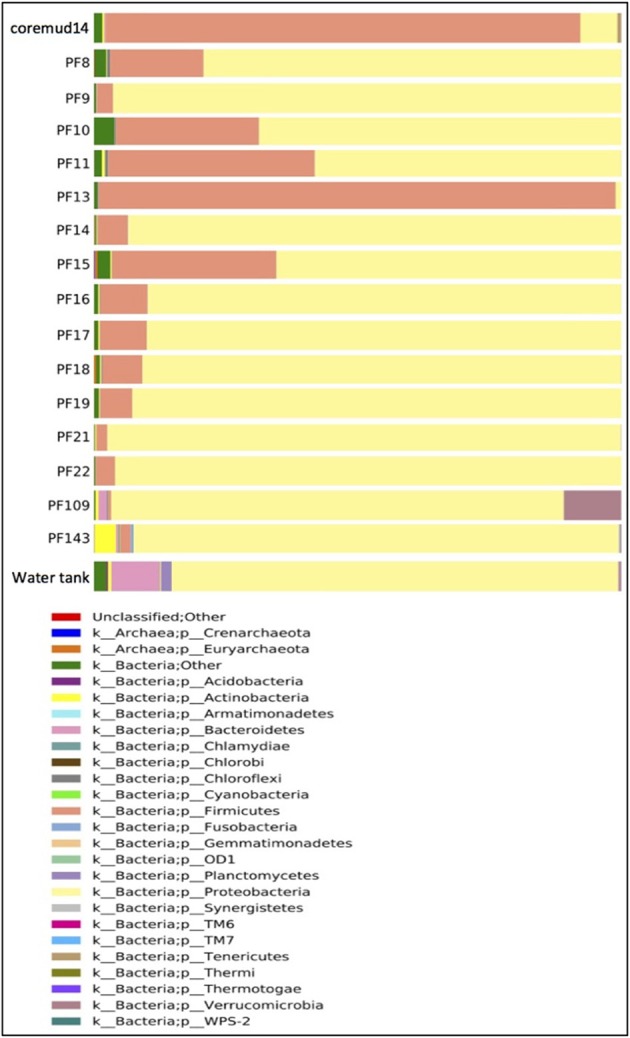
**Taxonomical summary of the Paaratte Formation at the phylum level**. Taxonomic data from the Ribosomal Database Project were used to classify each representative sequence from drilling mud (coremud14), a subset of pre-scCO_2_ and post-scCO_2_ injection water samples and a representative aliquot of water from surface storage tanks.

Taxonomical assignment of each representative sequence revealed a high abundance of *Firmicutes* in the drilling mud (90.0%) and PF13 (98.0%) communities. The remaining PF samples and water tank communities were overwhelmingly comprised of *Proteobacteria* (60.8–99.9%). *Crenarchaota, Euryarchaeota, Acidobacteria, Actinobacteria* and *Bacteroidetes* were amongst other phyla that accounted for a smaller percentage of the microbial community (Figure 3).

#### Genera summary

**Figure 4:**
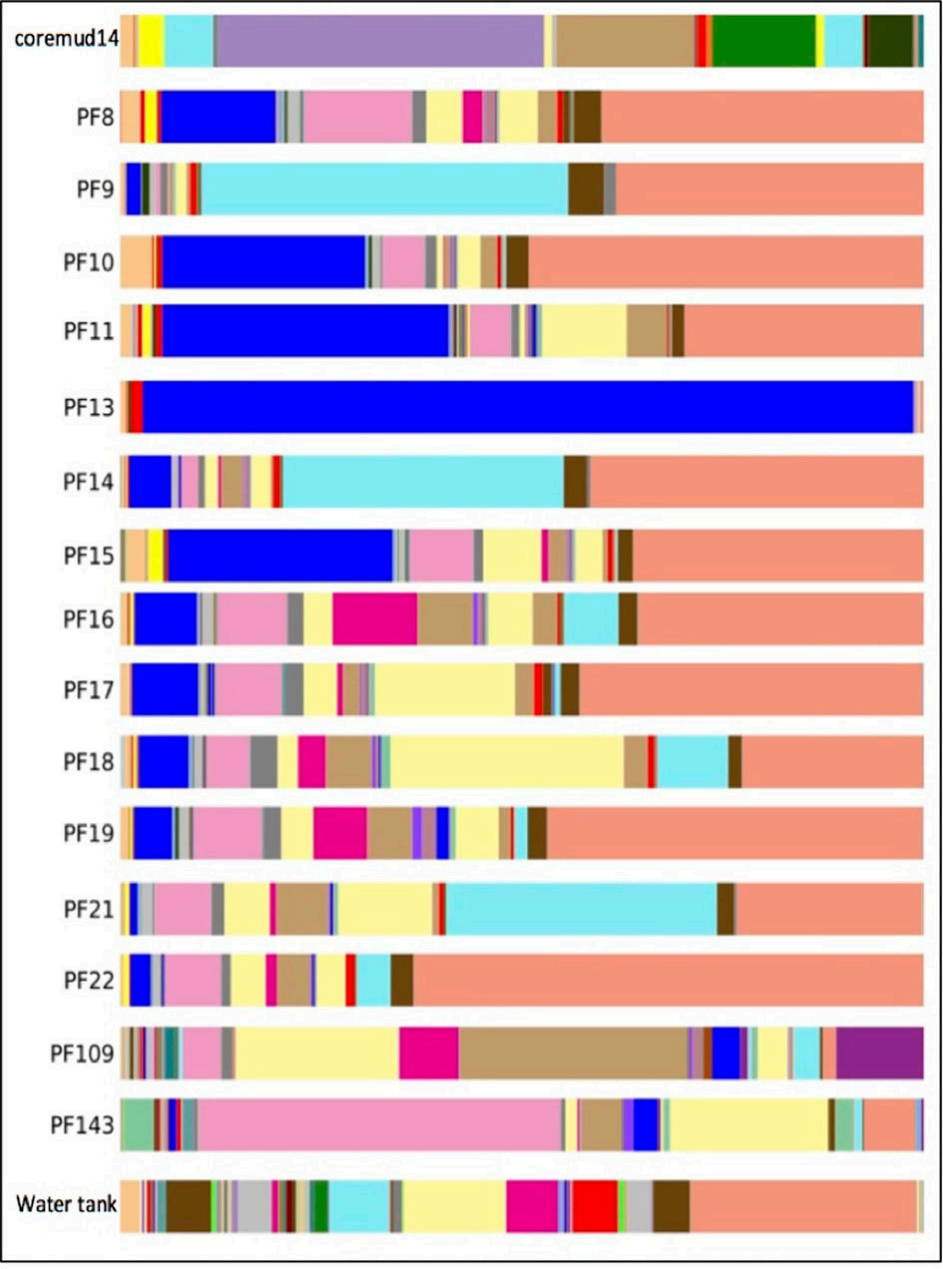
**Taxonomic summary of the Paaratte Formation at the genus level**. Taxonomic data from the Ribosomal Database Project were used to classify each representative sequence from drilling mud (coremud14), a subset of pre-scCO_2_ and post-scCO_2_ injection water samples and a representative aliquot of water from surface storage tanks. The corresponding figure legend is provided in Supplementary Figure 5.

The predominant OTU in PF13 were identified as *Firmicutes/Carboxydocella* (98.0%; phylum/ genus). This finding was not representative of the exogenous drilling mud or water tank microbial communities. The remaining pre- and post-scCO_2_ PF samples exhibited lower abundance scores of 0.2–35.7% for *Carboxydocella.* The *Proteobacteria* groups *Pseudomonas, Thauera, Acinetobacter, Sphingobium, Decholoromonas*, and *Comamonas* were amongst the closest related to environmental sequences, while, rare members (<1.5%) of the Paaratte Formation community include *Shewanella, Moorella*, and *Methylobacterium.* Some of the aforementioned taxa are known to reduce iron, degrade aromatic compounds and grow by reducing carbon compounds with one or more carbon atoms but no carbon-carbon bonds (Arnold et al., 1990; DiChristina et al., 2002; Drake and Daniel, 2004; Ruebush et al., 2006). Bioinformatic analyses revealed opposing abundance profiles for *Carboxydocella* and *Pseudomonas*, fluctuating relative abundances for *Dechloromonas* and *Acinetobacter*, and a proliferation in *Sphingobium* and *Comamonas* (Figure 4), over the course of the scCO_2_ geosequestration experiment.

#### Beta-diversity: UniFrac principal co-ordinate analysis plot

**Figure 5:**
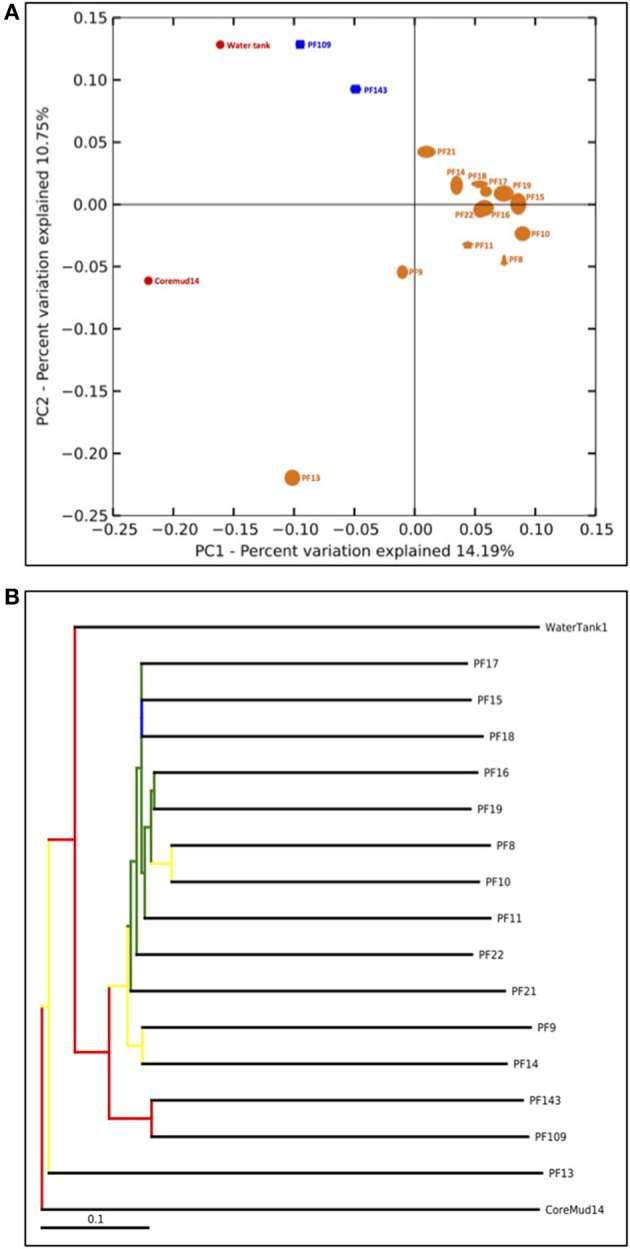
**Beta-diversity analyses of Paaratte Formation groundwater, drilling mud and storage tank microbial communities**. One thousand nine hundred and eighty-two sequences from each sample were selected at random and processed for a jackknifing analysis and represented as **(A)** a UniFrac Principal Co-ordinate Analysis plot according to its first two principal coordinates, and **(B)** a bootstrap-supported hierarchical clustering tree.

An assessment of beta-diversity (Figure 5A; diversity between samples) revealed a cluster of pre-scCO_2_ injection formation communities. The post-scCO_2_ injection communities also clustered together and in close association with the water tank community. Coremud14 (drilling fluid) and PF13 communities were distinct from the clustered nodes.

The detection of PEGs in PF12 may raise concerns of sample contamination. However, the isolation of Coremud14 community as represented on the PCoA plot (Figure 5A), and in further support, the microbial composition depicted at the genus level (Figure 4), indicate that the aquifer microbial communities were uncontaminated from the drilling fluid OTUs. Instead, we argue that the suite of operationally dependent peripherals (e.g., drilling fluid, U-tube engineering) are extensions of a subsurface ecosystem targeted for geological carbon sequestration, and must be considered as such to understand the overall microbial dynamics of the field-scale experiment.

#### Beta-diversity: UPGMA tree

The bootstrap-supported hierarchical tree (Figure 5B) illustrated the clustering of formation water sample communities away from the exogenous water tank and drilling fluid communities. Furthermore, post-scCO_2_ injection communities were more closely related to each other relative to pre-scCO_2_ injection communities.

#### Phylogenetic tree with associated metadata

**Figure 6:**
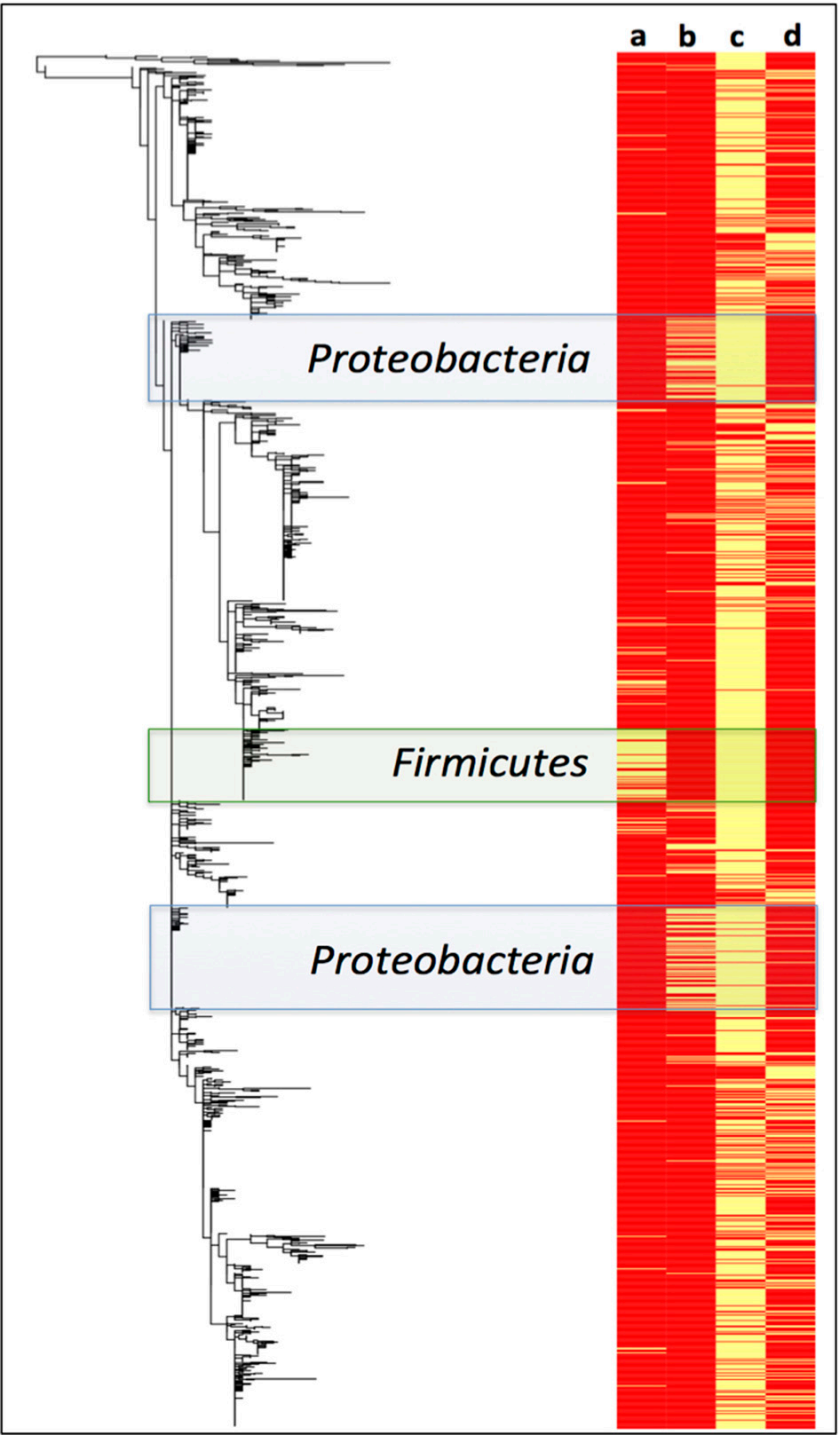
**Maximum likelihood phylogenetic tree of Paaratte Formation sequences displayed with its metadata**. Yellow bars on the heatmap indicate that the associated external node is an OTU that has been described to carry the genes associated with the expression of a (a) carbon monoxide dehydrogenase (CODH; green highlight), and a (b) polyethylene glycol dehydrogenase (PEGDH; blue highlight). Metadata also indicate if the external node is associated with the (c) pre-scCO_2_ injection phase or the (d) post-scCO_2_ injection phase. PyNAST aligned sequence data were used to construct the phylogenetic tree using the Maximum Likelihood algorithm on MEGA5.

A Maximum Likelihood phylogenetic tree of Paaratte Formation sequences was generated to obtain greater resolution of phylogenetic relationships to known cultivated or uncultured environmental microorganisms. Metadata associated with the ML tree of Paaratte Formation sequences (Figure 6) illustrated the separate clustering of external nodes that have been described to carry the genes responsible for the expression of either carbon monoxide dehydrogenase (CODH) or polyethylene glycol dehydrogenase (PEGDH) activities. These external nodes clustered in such a way that they revealed a predominant association with sequences derived from the pre-scCO_2_ injection phase.

## Discussion

Previous microbial community analyses focused on understanding the microbiota of potential geosequestration reservoirs prior to CO_2_ injection, or *in vitro* studies using experimental reaction media and representative geological materials (Basso et al., 2009; Dupraz et al., 2013; Kirk et al., 2013). In contrast, this study elucidated the dynamics of *in situ* microbial community structure changes associated with scCO_2_ stress in a deep subsurface aquifer during a field-scale geosequestration experiment.

Sampling via the U-tube system, as described previously (Freifeld, 2005, 2009), allowed for isolation of groundwater samples for geochemical and microbial analyses under *in situ* conditions without compromising the physical or chemical stability of the injected scCO_2_ plume or excess dissolved CO_2_ species deriving from the scCO_2_. A suite of geochemical and microbial analyses began at sample PF8 when drilling fluid-derived fluorescein levels declined to 2 × 10^−2^ ppm and groundwater was hence considered relatively pristine at the time of the experiment. However, further analysis of formation water samples by gas chromatography mass spectrometry of low molecular weight fatty acid compounds revealed that PF12, PF167, and PF221 samples contained polyethylene glycols (PEGs) interpreted as having derived from synthetic residual drilling fluid. These PEGs were below the level of detection in sample PF16, and this sample was therefore considered as geochemically “pristine” during the experimental phase prior to the injection of scCO_2_.

Poly-carbon compounds, such as propionate and butyrate appeared in both PF12 and PF16 samples (Figure 2). However, relative abundances of propionate and butyrate were 100 to 1000 times higher in sample PF12. Both organic compounds are well known products of microbial fermentation reactions, with the former being an intermediate of pyruvate and lactate fermentation (Schultz and Weaver, 1982; Janati-Idrissi et al., 1989). The observed decrease in both PEGs and poly-carbon compound concentrations from PF12 to PF16 indicates a probable temporal shift in microbial community (Figure 4) from predominantly fermentative organic degradation pathways of drilling fluid-derived residual PEG compounds to an increasing potential for respiratory degradation of the biodegradation products of fermentative reactions, all occurring within the pre-scCO_2_ injection phase. Propionate is an organic carboxylic acid that is biologically produced as its coenzyme A ester from metabolic breakdown of fatty acids (Schink, 1984). This compound registered approximately 500 times greater concentration in PF221, a sample obtained after both scCO_2 and organic tracer injections, in comparison to the levels recorded as residual from drilling fluid-contaminated samples. Furthermore, the absence of C_4+_ fatty acids in the neat organic tracers supports the interpretation that the presence of propionate in PF221 is therefore a by-product of microbial metabolism during the post-scCO_2_ injection phase, which suggests that microbial activity within the Paaratte Formation continued after scCO_2_ injection. This activity post-scCO_2_ injection indicates a persistence of the aquifer microbial biosphere under scCO_2_ stress, and substantiates the need for understanding potential microbially-driven impacts on the cycling and long-term sequestration of anthropogenic scCO_2_.

The overall microbial community structure of the Paaratte Formation, as assessed by whole community 16S rRNA gene profiling, shifted significantly toward the increasing dominance of *Proteobacteria* from an initial community structure predominated by *Firmicutes* during the pre-scCO_2_ injection phase (from 2% in PF13 to as high as 97% in PF21). This observed transformation of the aquifer microbial community composition corresponded to the observed temporal shift in inferred metabolic potential, from the capacity for fermentative degradation of residual drilling fluid organic compounds to predominantly species associated with respiratory heterotrophy, as propionate and butyrate disappeared from samples PF12 to PF16.

The microbial diversity index of the Paaratte Formation groundwater prior to scCO_2_ injection, and the subsequent “first order” change post-injection to support the proliferation of two groups, *Comamonadaceae* and *Sphingomonadaceae*, implies that a temporal shift in microbial community structure occurred in response to scCO_2_ injection. Furthermore, “second order” changes in the “background” microbial community prior to scCO_2_ injection reflected responses to organic geochemical conditions associated with the scCO_2_ injection experiment. These responses included microbial utilization of PEG, which can be degraded by dehydrogenase enzymes. In fact, membrane-bound PEG-dehydrogenases (PEGDH) were originally purified from *Sphingomonas* spp., (Sugimoto et al., 2001), while alcohol dehydrogenases found in *Comamonas testosteroni*, *Cm. acidovorans* and some *Pseudomonas* spp., have been shown to be capable of degrading PEGs (Obradors and Aguilar, 1991; De Jong et al., 1995; Stoorvogel et al., 1996; Kawai, 2002). The distribution of OTUs associated with the potential for PEG degradation is ubiquitous among environmental microbiota (Marchal et al., 2008), and extends to the current study where *Comamonas, Pseudomonas* and Sphingomonads were detected (Figure 4).

The subsistence of the predominant *Firmicutes* genus, *Carboxydocella*, throughout the pre- and post-injection phases, suggests that it may be an important constituent of the Paaratte Formation native microbiota. *Carboxydocella* is an anaerobic thermophile that grows optimally at the *in situ* temperature of the Paaratte Formation aquifer, which ranges from 55 to 60°C, and has been previously described to express a carbon monoxide dehydrogenase (CODH) enzyme (Sokolova, 2002). Homologs of *cdh*, the gene responsible for the expression of the CODH enzyme, outside what is described as the CODH/acetyl-CoA synthase (CODH/ACS) complex, indicates exogenous CO usage. However, regardless of whether there are multiple homologs in the genome or just the single *cdh* gene, this dehydrogenase autotrophically catalyzes the oxidation of CO to CO_2_, according to the equation:

(1) $$\text{CO}+{\text{H}}_{2}\text{O}\leftarrow \to {\text{CO}}_{2}+{\text{H}}_{2}$$

(Svetlitchnyi et al., 2001; Ragsdale, 2004; Oelgeschläger and Rother, 2008; Sokolova et al., 2009; Gullotta et al., 2011; Techtmann et al., 2011; Wilkins and Atiyeh, 2011).

An increase in CO_2_ activity through scCO_2_ injection could theoretically saturate the forward reaction of equation (1), and inhibit the oxidation of CO by a microbial CODH enzyme, resulting in increased and decreased levels of CO and H_2_, respectively, in the groundwater.

A survey of the aquifer microbial community for *cdh*, and the gene encoding the protein responsible for degrading residual drilling fluid organics, PEGDH, highlights distinct clustering of taxonomic units described to carry the genes involved in CO oxidation and PEG degradation (Figure 6). The clustering suggests that these enzymatic capabilities are not likely to be shared with any one group of OTUs. Furthermore, community 16S rRNA gene data revealed an inverse relationship of these OTUs in each of the 17 microbial communities analyzed (Figures 3, 4). The OTUs that are associated with *cdh* and PEGDH-encoding genes (or gene homologs) are more closely associated with 16S rRNA gene sequences recovered from groundwater samples obtained during the pre-scCO_2_ injection phase (Figure 6). Therefore, the disappearance of any of these taxonomic groups as a result of a CO_2_ geosequestration event could have downstream impacts on the microbial community and biogeochemical processes affecting the fate of injected CO_2_ (e.g., biomineralization and/or conversion into biomass). However, analysis of the metagenome is currently performed to provide further insight to the functional gene profile of the Paaratte Formation microbial community, and determine the presence/ absence of *cdh* genes.

In summary, the current study presents a field-scale, cultivation-independent investigation into the changes to the *in situ* microbial community dynamics of the Paaratte Formation after the injection of many kilotons of scCO_2_. Analysis of formation water sampled via the U-tube system revealed a community profile predominated by *Firmicutes* during the early stages of the CO_2_ geosequestration project before a shift towards mainly *Proteobacteria*. The temporal shift in taxonomic grouping corresponds to the shift from fermentative degradation of residual drilling fluid organics to mainly respiratory metabolism as inferred from the decline in relative peak concentrations of propionate and butyrate. The persistence of *Carboxydocella, Comamonadaceae* and *Sphingomonadaceae* after scCO_2_ injection suggests that these groups could adapt to the changes in groundwater chemistry resulting from the CO_2_ geosequestration experiment.

### Conflict of interest statement

The authors declare that the research was conducted in the absence of any commercial or financial relationships that could be construed as a potential conflict of interest.
