# Supplementary figures and images for: Changes in the deep subsurface microbial biosphere resulting from a field-scale CO2 geosequestration experiment
Source: Front Microbiol. 2014 May 14;5:209. doi: 10.3389/fmicb.2014.00209 (PMC4030138; doi:10.3389/fmicb.2014.00209)

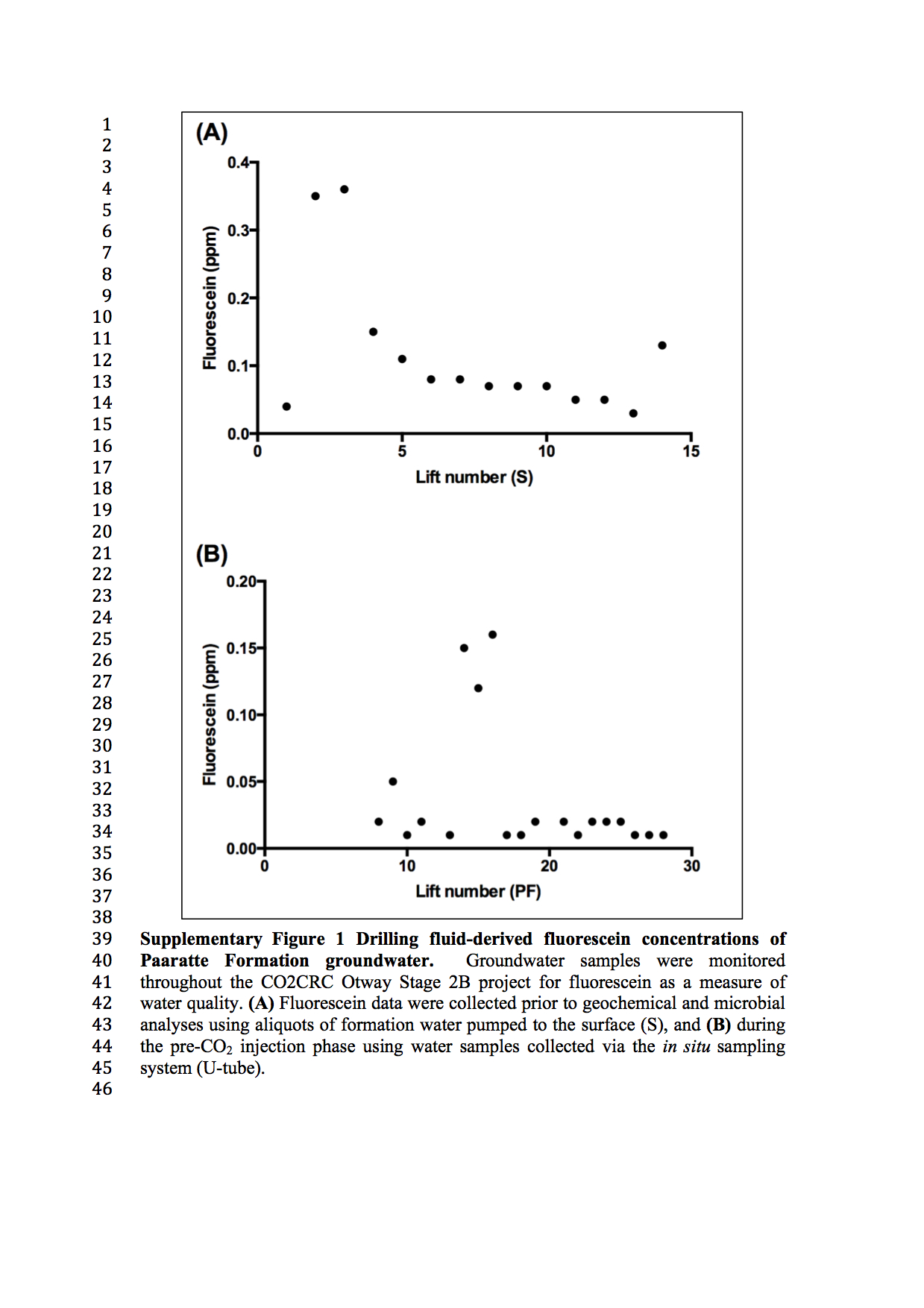

Supplement: Supplementary file 1 [file Presentation10.ZIP › Mu et al Supplementary Figure 1.jpg]

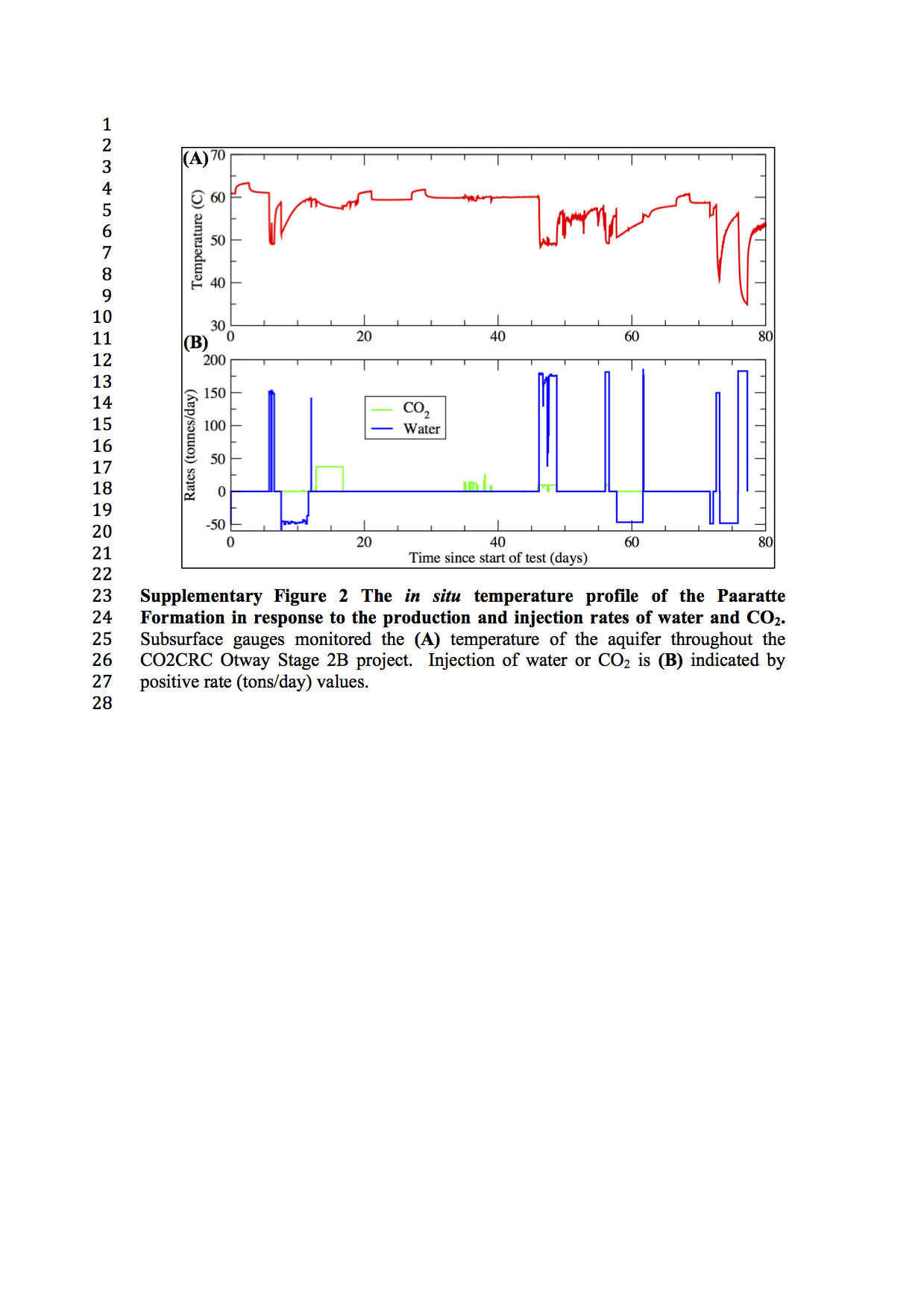

Supplement: Supplementary file 1 [file Presentation10.ZIP › Mu et al Supplementary Figure 2.jpg]

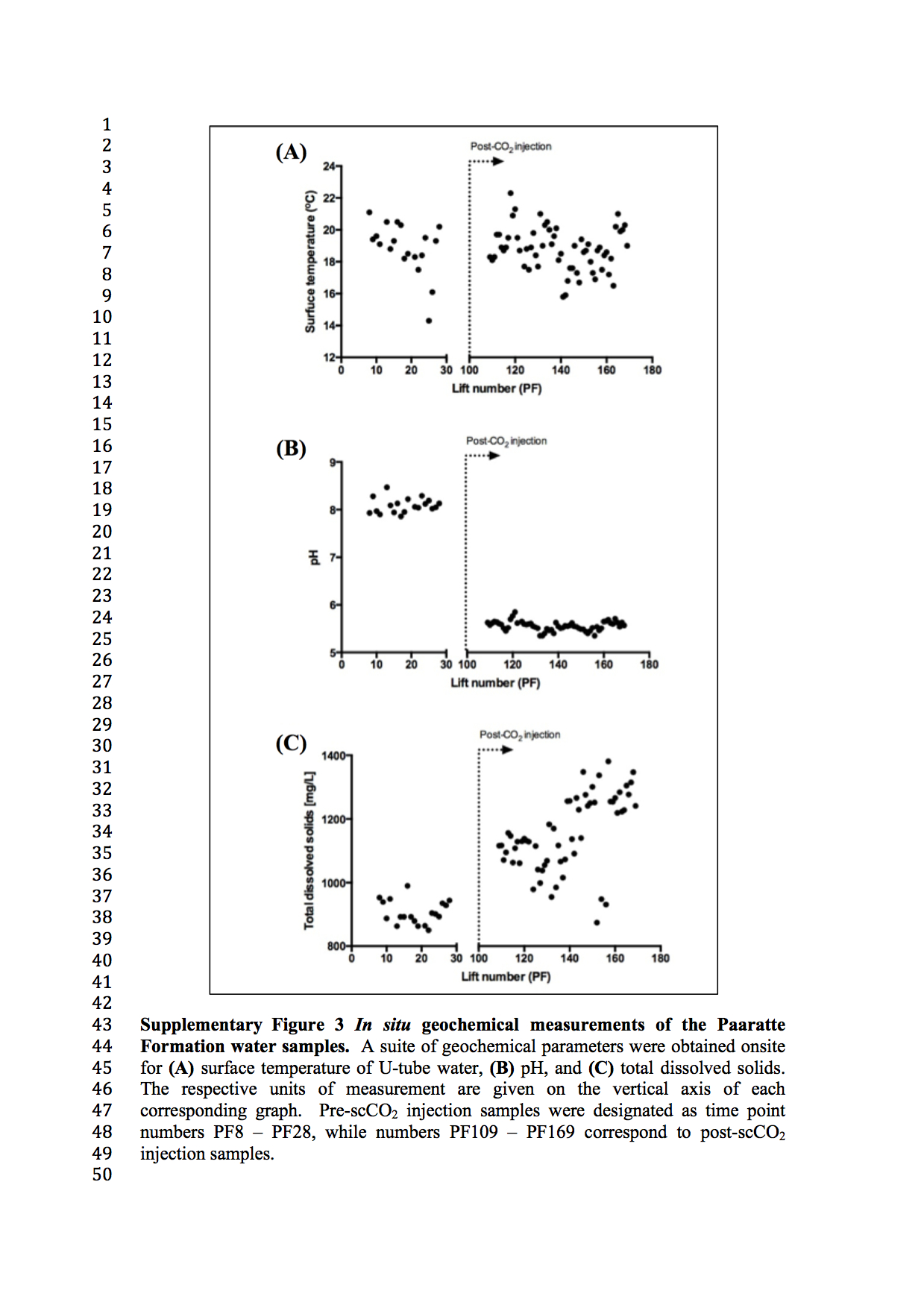

Supplement: Supplementary file 1 [file Presentation10.ZIP › Mu et al Supplementary Figure 3.jpg]

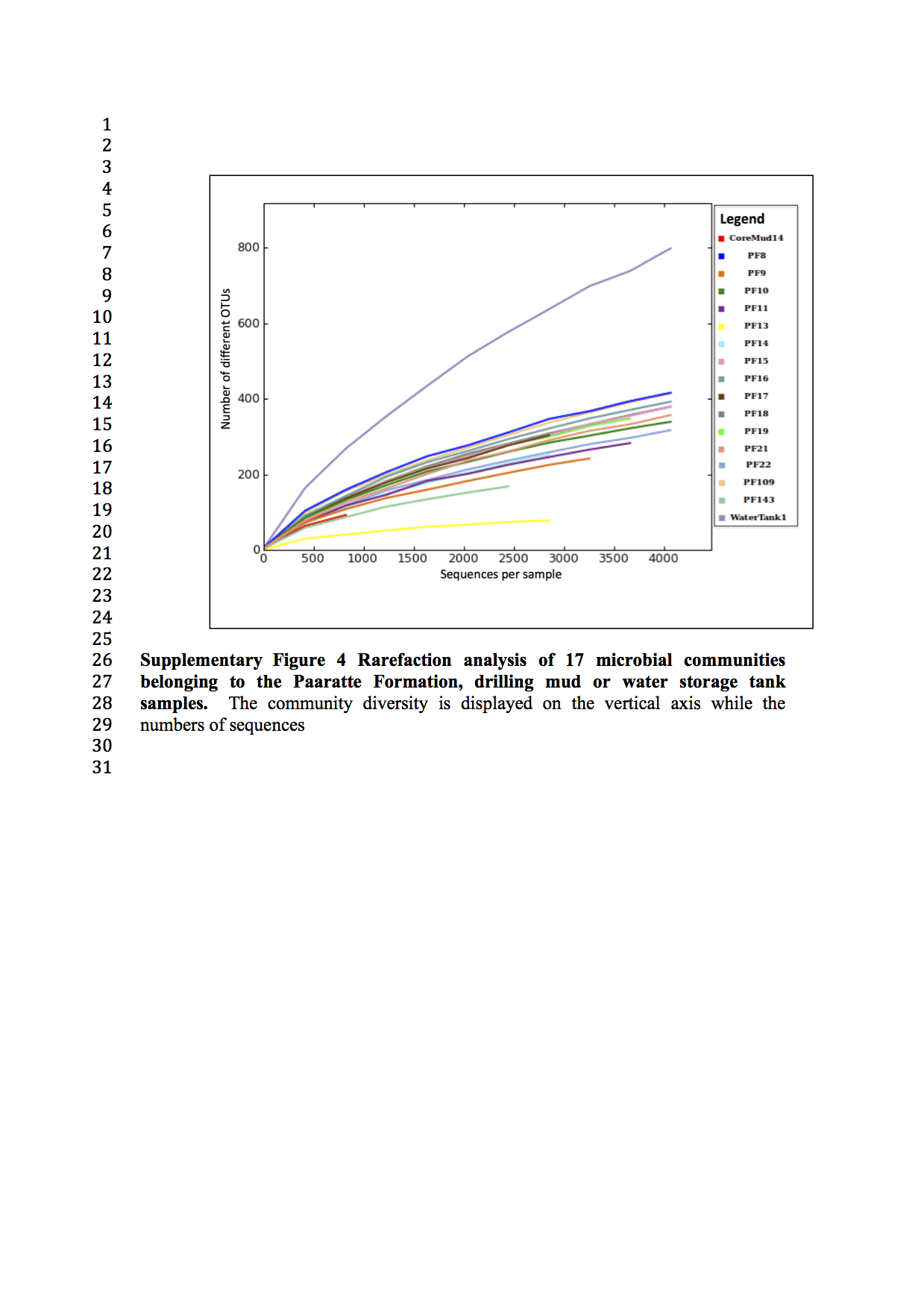

Supplement: Supplementary file 1 [file Presentation10.ZIP › Mu et al Supplementary Figure 4.jpg]

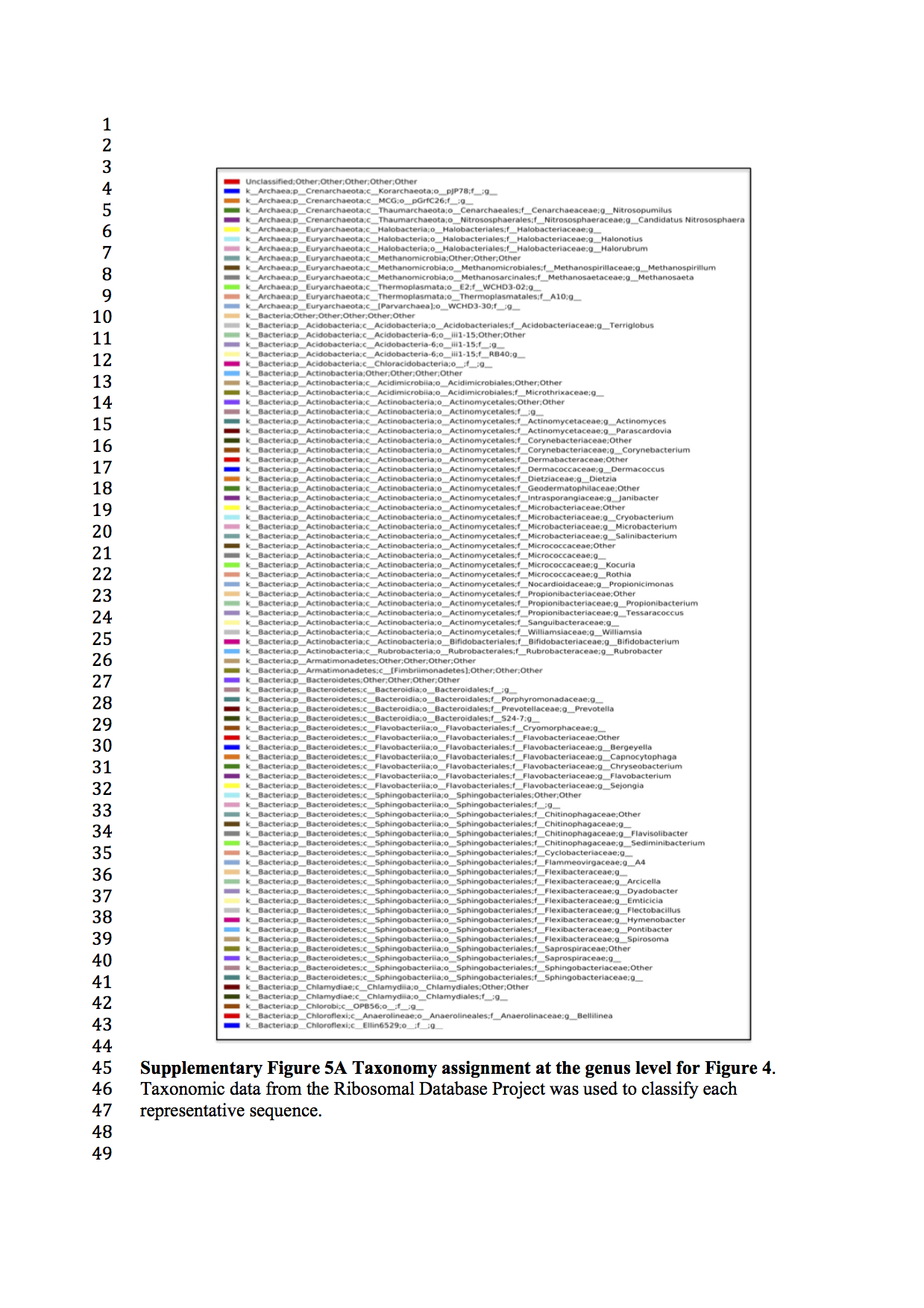

Supplement: Supplementary file 1 [file Presentation10.ZIP › Mu et al Supplementary Figure 5A.jpg]

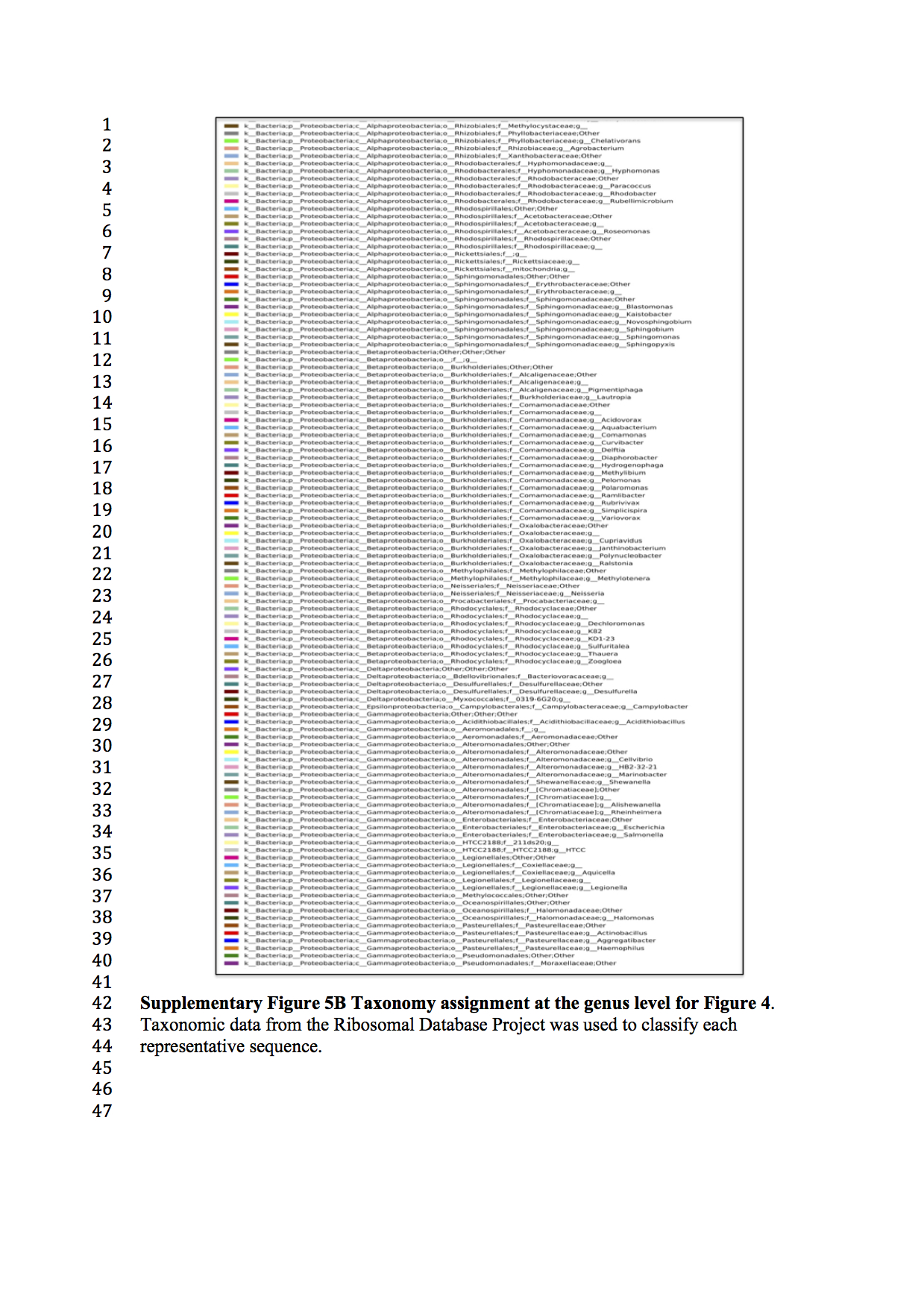

Supplement: Supplementary file 1 [file Presentation10.ZIP › Mu et al Supplementary Figure 5B.jpg]

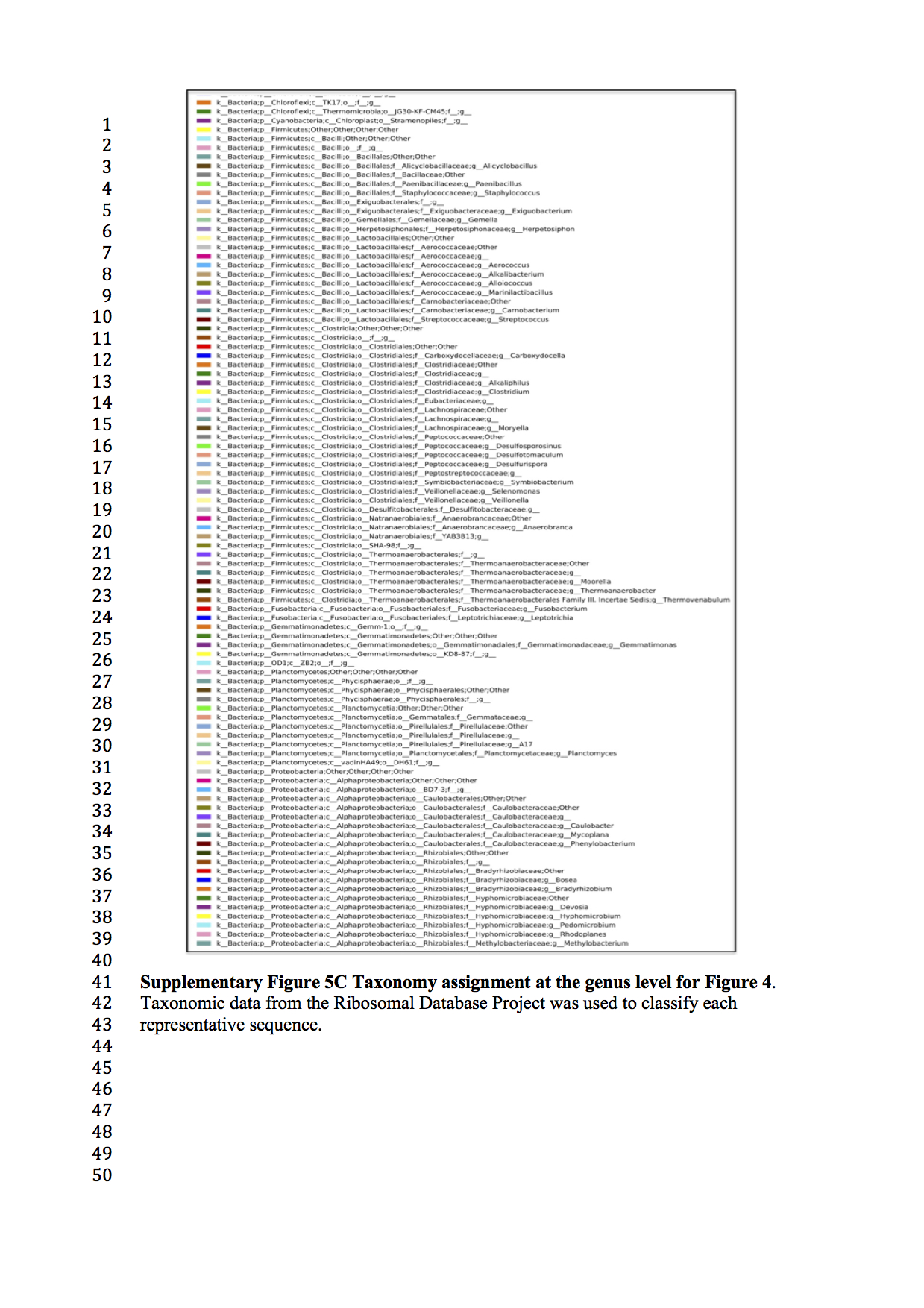

Supplement: Supplementary file 1 [file Presentation10.ZIP › Mu et al Supplementary Figure 5C.jpg]

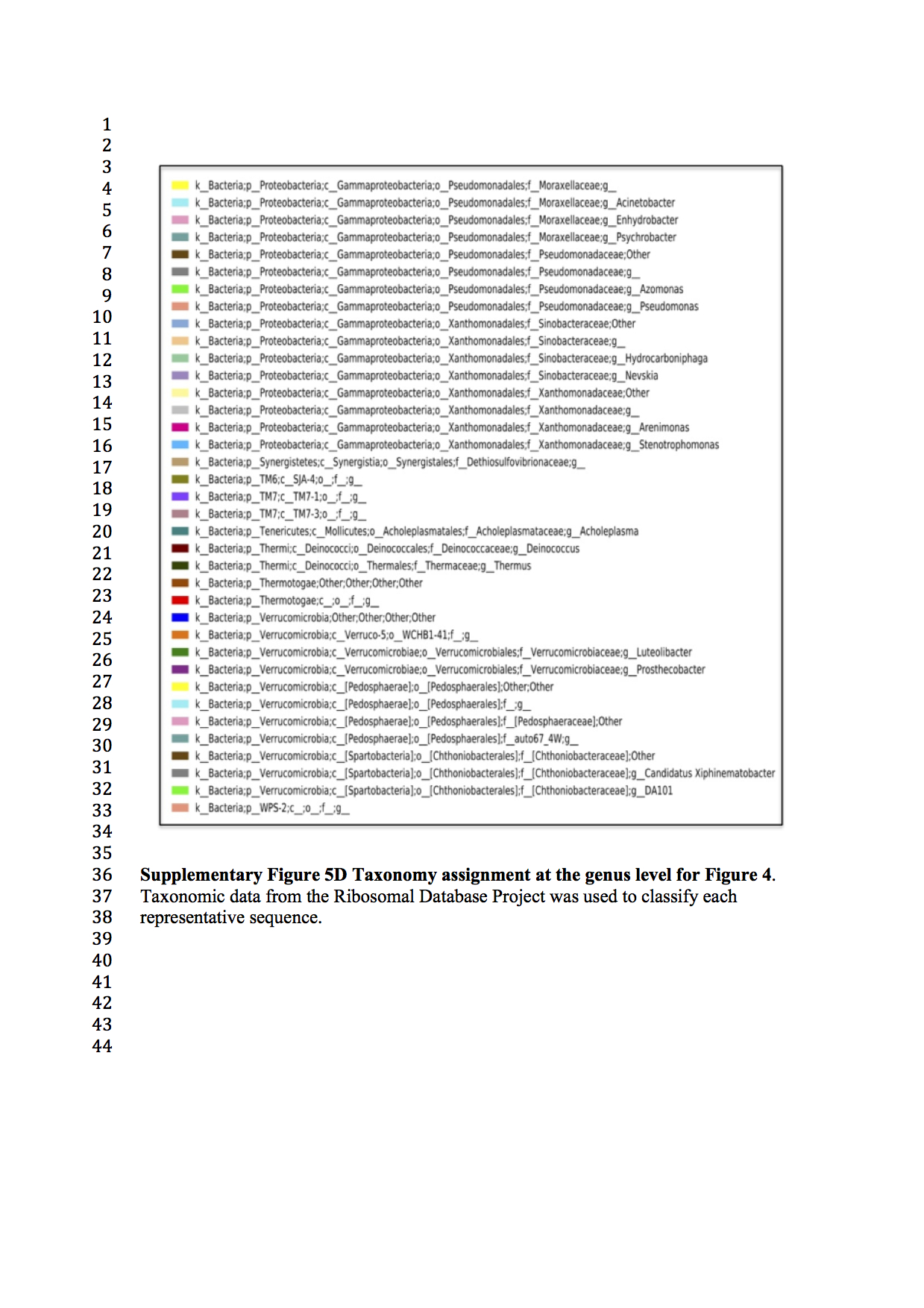

Supplement: Supplementary file 1 [file Presentation10.ZIP › Mu et al Supplementary Figure 5D.jpg]
